# Axillary Fibroadenoma: An Unusual Clinical Presentation

**DOI:** 10.7759/cureus.73888

**Published:** 2024-11-18

**Authors:** Maria L Rodrigues, Prathvi Shetty, Sueallen L D'Souza

**Affiliations:** 1 Department of General Surgery, Father Muller Medical College, Mangalore, IND; 2 Department of Pathology, Father Muller Medical College, Mangalore, IND

**Keywords:** axilla, axillary swelling, benign breast tumor, case report, ectopic breast tissue, fibroadenoma

## Abstract

Axillary swellings, particularly in women, often raise suspicion due to the high association of such swellings with breast carcinoma manifesting as metastatic lymph node involvement. Tuberculous lymphadenopathy is one of the primary diagnoses in India. Fibroadenomas are common benign breast tumors, but their occurrence in the axilla, arising from ectopic breast tissue (EBT), is rare and may present a diagnostic challenge. This report presents a case of a patient with axillary swelling, initially suspected to be isolated lymphadenopathy, which was later diagnosed as a fibroadenoma. The case highlights the clinical significance of swelling in the axilla, such as ectopic breast tissue, which undergoes physiological changes similar to those of normal breast tissue. Additionally, pathological changes can occur in this tissue, making diagnosis challenging. Swelling in the axilla can pose a diagnostic dilemma, and pathologies associated with ectopic breast tissue require careful consideration.

## Introduction

Fibroadenomas are common benign breast tumors, occurring in approximately 25% of cases, primarily in women aged 15-35. Approximately 2%-6% of women have ectopic breast tissue (EBT), but fibroadenoma arising from it is rarely documented [1]. A fibroadenoma typically presents as a small, well-defined, painless, mobile mass, often detected upon palpation [1,2]. Ultrasound serves as the first-line imaging modality for axillary swelling, yet histopathological evaluation remains essential for confirming the diagnosis, and straightforward excision is the standard line of treatment [1]. This case report describes an unusual presentation of fibroadenoma in the axillary region and the diagnostic dilemma it caused.

## Case presentation

A 35-year-old woman presented with a painless swelling in her right axilla, which she had noticed over the past two years. She reported no associated symptoms, such as fever, weight loss, or nipple discharge. Physical examination revealed a firm, non-tender, mobile swelling measuring 3 × 4 cm in the right axilla. The patient had no significant medical or family history. The differential diagnoses considered were lymphadenopathy, lipoma, or fibroma.

**Figure 1:**
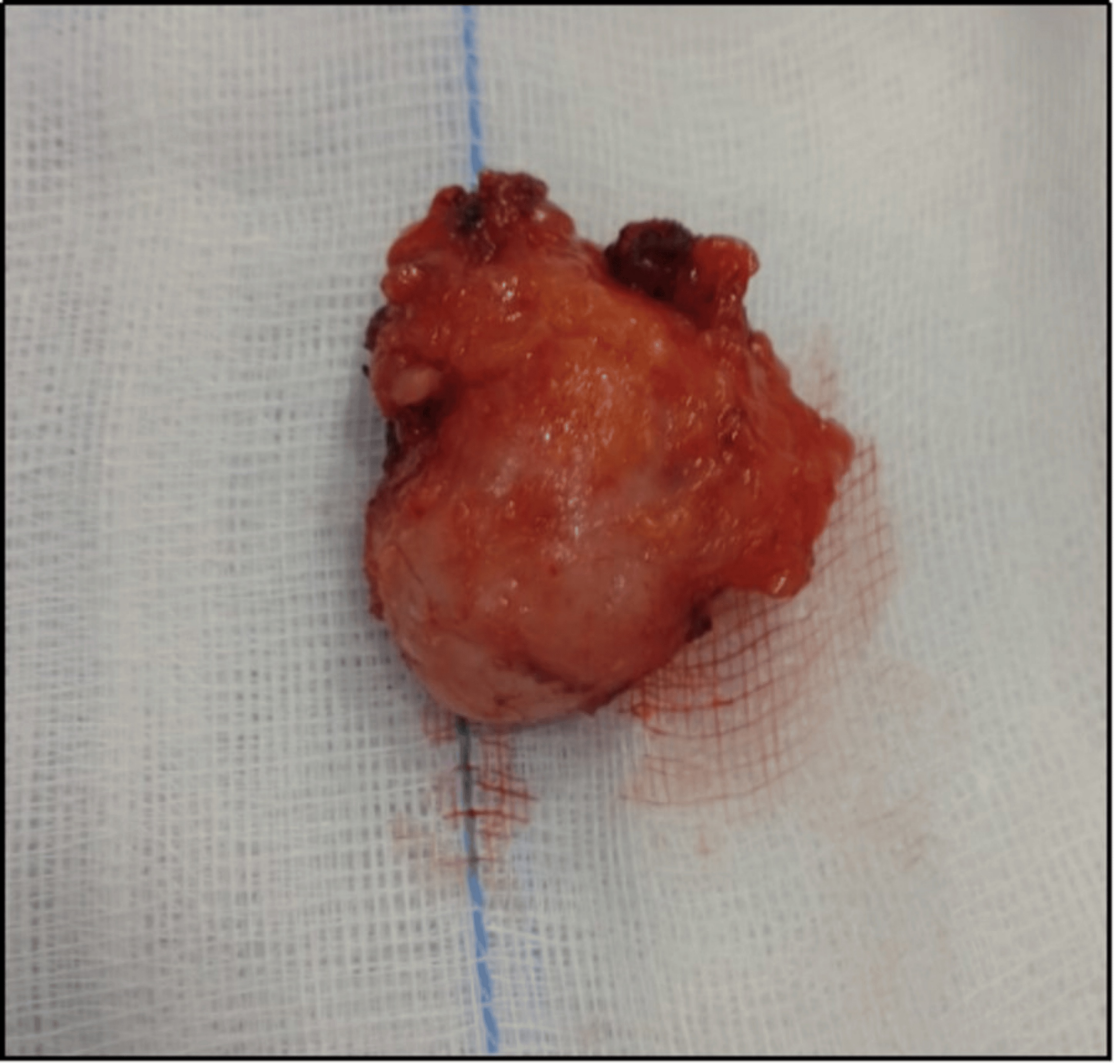
Gross specimen of the excised fibroadenoma

Her hematological investigations were within the normal limits. A bilateral breast sonography report revealed an ovoid hypoechoic solid lesion of 3.1 × 1.2 cm in the right axilla, likely fibroadenoma. Complete excision of the swelling was carried out to establish a definitive diagnosis and provide treatment. A firm, well-circumscribed mass measuring 3.8 × 2.5 × 2 cm was excised via an axillary incision (Figure 1) and subsequently sent for histopathological examination.

**Figure 2:**
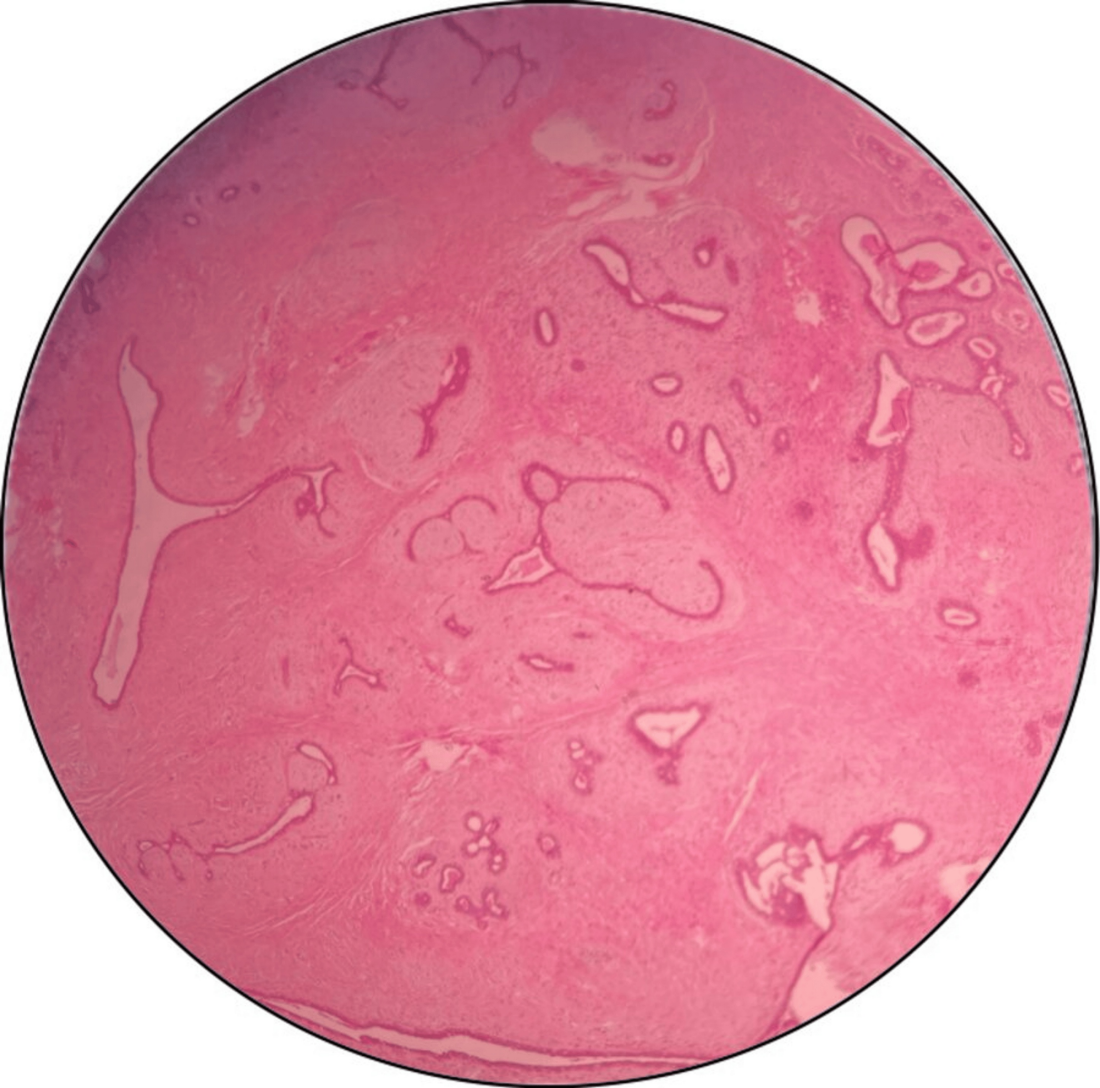
Microscopic findings of the surgical specimen Proliferation of bilayered ducts lined by epithelial and myoepithelial cells showing both pericanalicular and intracanalicular growth patterns set in a fibromyxoid stroma (H&E staining, original magnification: 10×) H&E: hematoxylin and eosin

**Figure 3:**
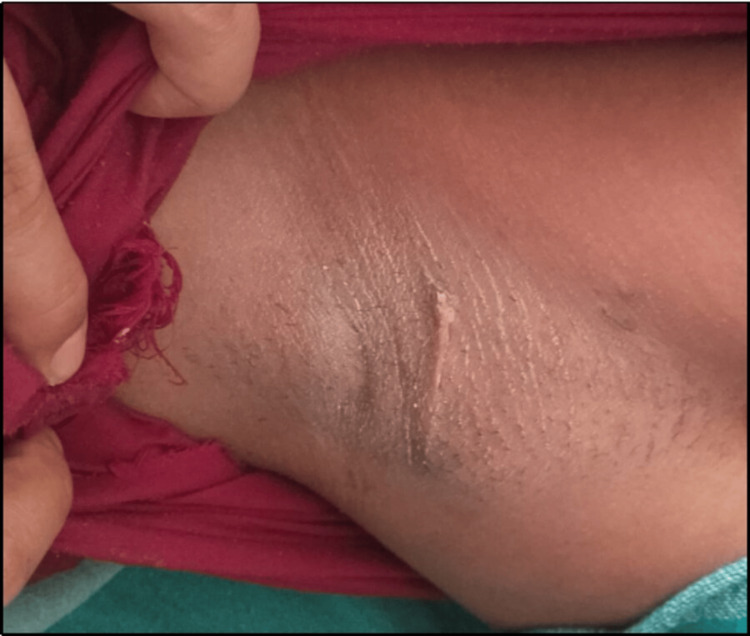
Location of the axillary fibroadenoma and healed scar postoperatively

The histopathological report confirmed the diagnosis of fibroadenoma, showing typical features of stromal and epithelial proliferation without atypia (Figure 2). Postoperatively, the patient had a good recovery, and the scar healed by primary intention (Figure 3).

## Discussion

Ectopic breast tissue (EBT) is characterized by the occurrence of breast tissue (glandular tissue, nipple-areola complex, or both) in locations outside the typical pectoral region. EBT predominantly manifests throughout the milk line, extending from the mid-axilla to the inguinal region [3]. That said, it has been known to occur in less common areas such as the scapula, axilla, thigh, and vulva, with the axilla being the frequently observed location. The prevalence of EBT in women is estimated to range from 0.4% to 6%, with higher rates reported among Asian women, as well as rare associations with other diseases. Similar to normal breast tissue, EBT is susceptible to various conditions, including fibrocystic alterations, breast inflammation (mastitis), ductal papillomas, abscesses, fibroadenomas, malignant tumors (carcinomas), and phyllodes tumors [3,4].

During early embryonic development, milk lines form as ectodermal thickenings along the embryo's sides. Typically, most of these ridges disappear, with the exception of two areas in the chest, that differentiate into breasts. Ectopic breast tissue can occur if any part of the mammary ridge does not regress [3].

Changes in estrogen and progesterone levels during puberty, pregnancy, and the menstrual cycle are key factors in the development of fibroadenomas, potentially promoting their growth in breast tissue [5]. Ectopic breast tissues are considered to enlarge and lead to discomfort in response to hormonal changes [3].

Kajava proposed a framework for the classification of ectopic breast tissue, which includes the following: Class I, a fully developed breast containing the nipple, areola, and glandular tissue; Class II, contains nipple and glandular tissue but lacks the areola; Class III, has areola and glandular tissue, but the nipple is absent; Class IV, only glandular tissue is present; Class V, the nipple and areola are present, but there is no glandular tissue (referred to as pseudomamma); Class VI, presence of additional nipples (polythelia); Class VII, only the areola is present (polythelia areolaris); and Class VIII, just a patch of hair is present (polythelia pilosa) [3,6]. Our case was classified as Class IV.

The possible diagnoses when a mass is found in the axillary region include conditions such as enlarged lymph nodes, benign fatty tumors (lipomas), tuberculosis, nerve growths (neuromas), the extension of breast tissue toward the axilla (axillary tail of Spence), skin growths including epidermal inclusion cysts and sebaceous cysts, chronic inflammatory skin condition (suppurative hidradenitis), and blood vessel abnormalities and lesions originating from ectopic breast tissue [3]. EBT can develop conditions similar to normal breast tissue, with carcinoma being the most common. Thus, recognizing a swelling along the milk line as EBT is vital for early detection and diagnosis of related diseases [3,4]. Comprehensive imaging and histopathological assessment are crucial for distinguishing these conditions [3].

Lee's retrospective study assessed 39 patients diagnosed with fibroadenoma in the axilla. A majority of patients sought evaluation due to concerns about an axillary mass, fearing potential malignancy. Each patient had the excision performed through an axillary incision and reported high levels of satisfaction with both the resolution of symptoms and the cosmetic outcome [7].

The larger range of differential diagnoses can delay the identification of malignancy arising from ectopic breast tissue, contributing to a less favorable outcome. Furthermore, ectopic breast tissue in patients may be accompanied by renal abnormalities, as the genitourinary system and mammary structures develop concurrently. This association is an important consideration during patient evaluation [1].

In this case, the patient likely did not notice the ectopic breast tissue initially and may have mistaken it for a pad of fat. It only became apparent when it developed into a fibroadenoma, appearing as a well-circumscribed mass. The axillary tail of Spence is an extension of the breast that originates from the lateral side of the thoracic breast and is situated within the deeper part of the axilla [3]. However, in this patient, the axillary mass was distinct from the breast and positioned superficially, which confirmed that it was ectopic breast tissue and not an extension of breast tissue.

## Conclusions

This case emphasizes the need to include fibroadenoma in the differential diagnoses when evaluating axillary masses. Although rare, fibroadenomas can occur in ectopic breast tissue, and accurate diagnosis relies on thorough imaging and histopathological evaluation. Awareness of such presentations can aid clinicians in providing appropriate management and avoiding misdiagnosis.
